# A Case of Advanced Bilateral Inflammatory Breast Cancer: A Radiological Perspective

**DOI:** 10.7759/cureus.34145

**Published:** 2023-01-24

**Authors:** Ryan Salmanzadeh, Kristopher Aghemo, Babitha Thatiparthi, Seema A Al-Shaikhli, Austin Salmanzadeh, Osmany DeAngelo, David Martin, Suporn Sukpraprut-Braaten

**Affiliations:** 1 Radiology, Larkin Community Hospital, Miami, USA; 2 Radiology, Herbert Wertheim College of Medicine at Florida International University, Miami, USA; 3 Biology, University of Central Florida, Orlando, USA; 4 Medical Education, Unity Health, Searcy, USA

**Keywords:** breast imaging, pet-ct, ct, metastatic breast cancer, inflammatory breast cancer

## Abstract

Inflammatory breast cancer (IBC) is a rare and aggressive form of breast cancer that accounts for only a small percent of invasive breast cancers in the United States. We report a case of advanced bilateral IBC in a 60-year-old female. This case report explores the clinical presentation, pathological findings, and different imaging modalities that can assist in the diagnosis of this disease. The initial diagnosis was based on imaging findings from both contrast-enhanced computed tomography (CECT) and positron emission tomography-computed tomography (PET-CT). The diagnosis was then confirmed with histopathological findings.

## Introduction

Inflammatory breast cancer (IBC) is a rare disease accounting for only 2% of all invasive breast cancer in the United States, often featuring advanced disease at the time of diagnosis, rapid progression, and an ominous prognosis [1]. Contralateral involvement is less commonly diagnosed, occurring in only 1%-5% of patients with primary IBC [2]. IBC is a poorly understood disease with little evidence regarding specific risk factors [3]. Lymphatic obstruction mimics inflammation by sometimes causing warmth, tenderness, and engorgement of the breast. These skin findings, along with rapid progression and late staging at the time of discovery, are characteristic of IBC [4]. Diagnosis is clinical and based on the aforementioned findings in conjunction with radiological and pathological evidence. Unfortunately, inflammatory breast cancer does not yet have strict diagnostic criteria, thus necessitating the compilation of more clinical data. With such limited data available, unique findings in cases of IBC are of high clinical value. This case demonstrated an atypical presentation, with extensive bilateral breast involvement only discovered incidentally. This paper will demonstrate a case of bilateral IBC and explore multidisciplinary findings with a focus on radiological imaging.

## Case presentation

**Figure 1:**
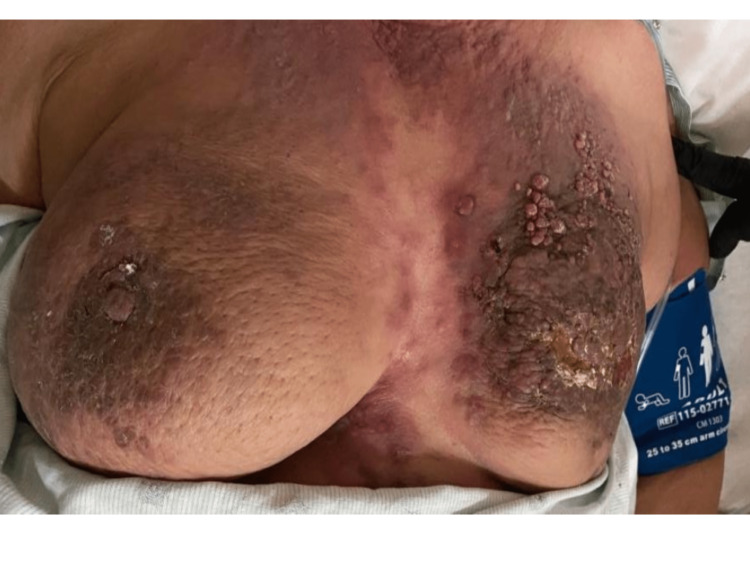
Erythematous, tender breasts with peau d'orange appearance due to dermal lymphatic invasion by tumor emboli. The left breast demonstrates more extensive nodular skin thickening, hyperpigmentation, nipple retraction, and frank ulceration from underlying tumor mass.

A 60-year-old female presented to the emergency department with right-sided hip pain. Physical examination demonstrated bilateral, asymmetric, tender, immobile breast masses with nipple retraction, peau d'orange appearance, skin hyperpigmentation, and frank ulceration (Figure 1). Bloody nipple discharge was present on the left breast. Palpable nodules were present in the bilateral axillary region. In addition, innumerable superficial verruca-like skin nodules were found involving both breasts, extending to the supraclavicular region and to the periumbilical region in the mid-abdominal wall (not shown).

**Figure 2:**
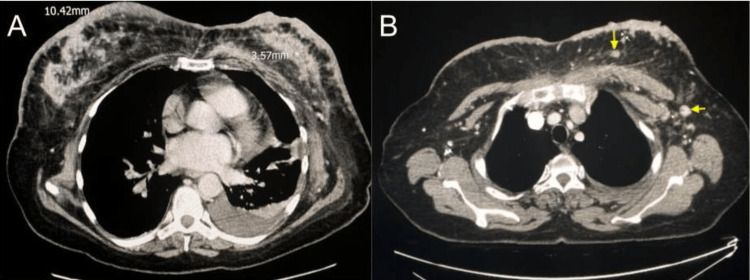
CECT of the chest (axial view). (A) Diffuse irregular skin thickening with dermal lymphatic extension and axillary adenopathy, asymmetric bilateral heterogeneously enhancing breast masses with spiculated borders and subtle surrounding fatty stranding, and innumerable metastatic nodules with the replacement of normal fatty hilum that conform to the fibroglandular tissue and axillary nodal stations. There is a central dystrophic calcification within the left breast mass with adjacent infiltration along the anterior chest wall, reticular thickening of the pectoralis musculature, and a malignant left pleural effusion. (B) Enhancing axillary adenopathy, level 1 and level 2 nodes on the left. CECT: contrast-enhanced computed tomography

**Figure 3:**
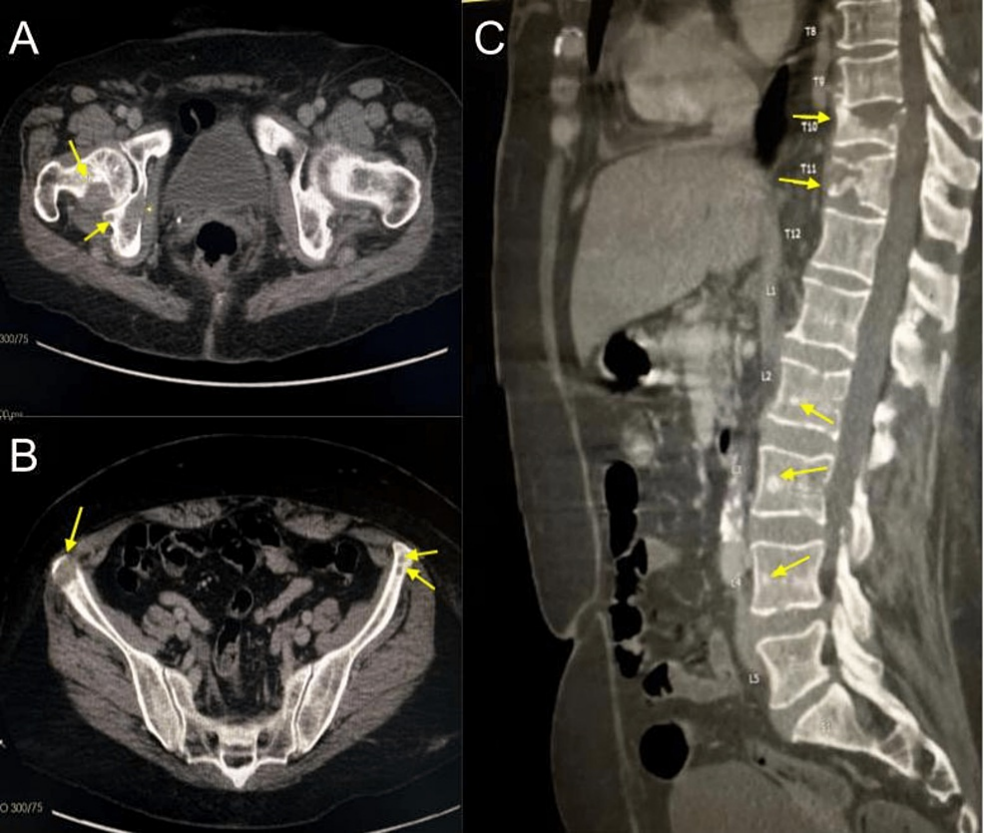
(A) CECT of the pelvis (axial view): destructive lytic lesions in the right femoral head and posterior column of the right acetabulum with an underlying pathological fracture (not shown). (B) CECT of the pelvis (axial view): bilateral lytic lesions involving the anterior iliac crests, expansile on the left. (C) CECT of the pelvis (sagittal view): diffuse bony metastases with multiple lytic and blastic lesions throughout the thoracolumbar spine. Pathological fracture seen at T10 with mild vertebral height loss. CECT: contrast-enhanced computed tomography

Initial pelvic X-ray demonstrated multiple lytic lesions. This prompted further investigation with contrast-enhanced computed tomography (CECT) imaging. CECT of the chest revealed bilateral severe diffuse thickening of the skin overlying both breasts associated with extensive infiltrative fibroglandular densities and nipple retraction, findings supportive of diffuse inflammatory breast disease (Figure 2A). Pathologically enlarged metastatic left-sided axillary lymphadenopathy and multiple small scattered breast masses in the upper inner quadrant of the left breast were also observed (Figure 2B). CECT of the pelvis demonstrated multiple lytic lesions in the bony pelvis (Figure 3A) and the right femur (Figure 3B). Multiple mixed lytic and blastic lesions to the thoracolumbar spine were also observed (Figure 3C).

**Figure 4:**
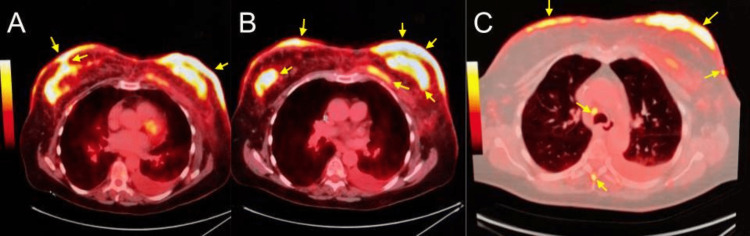
PET-CT of the chest (axial view). (A) Infiltrative FDG-avid left breast mass involving substantially the entire breast parenchyma. FDG-avid thickening of the skin involving the entire left breast. Somewhat less-extensive infiltrative mass involving the entire right breast parenchyma with nipple retraction. Diffuse FDG-avid skin thickening of the right breast. (B) Multiple FDG-avid axillary lymph nodes bilaterally, more extensive on the left. No FDG-avid pulmonary masses. FDG-avid moderate-size left pleural effusion. No FDG-avid left lower lobe consolidation, likely representing compressive atelectasis. (C) Bilateral subcutaneous breast tissue uptake with metastatic infiltration of the left pectoralis major muscle (better seen in B), left lower paraesophageal lymph node, and mid-thoracic vertebral arch. PET-CT, positron emission tomography-computed tomography; FDG, fluorodeoxyglucose

**Figure 5:**
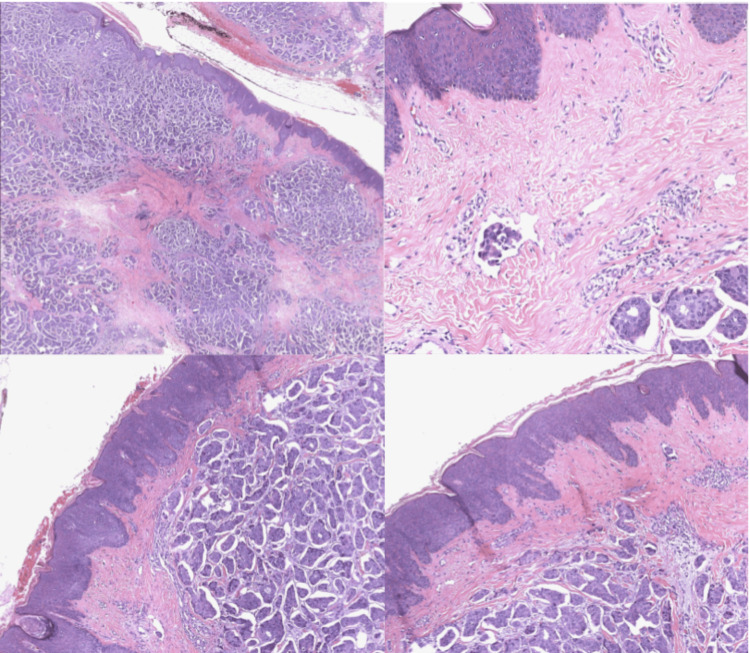
Histology of infiltrating ductal carcinoma involving dermal skin, including scattered dermal lymphovascular invasion.

A positron emission tomography-computed tomography (PET-CT) demonstrated infiltrative bilateral breast masses, axillary adenopathy, and diffuse bony metastasis, supporting the previous CECT findings. The PET-CT revealed multiple fluorodeoxyglucose (FDG)-avid bone lesions present throughout the spine, pelvis, sacrum, and proximal appendicular skeleton. Metastatic adenopathy was identified in the left supraclavicular and bilateral axillary lymph nodes (Figure 4A-4C). Bilateral breast pathology revealed triple-positive infiltrating ductal carcinoma with dermal lymphatic involvement (Figure 5). After a review of the clinical, radiological, and histological evidence, a diagnosis of stage IV bilateral inflammatory breast cancer was established.

## Discussion

Although it contains the word "inflammatory" in its name, IBC does not involve a true inflammatory process. Tumor emboli often obstruct the dermal lymphatics, leading to the classic dimpled skin appearance, commonly described as peau d'orange, which is present in 75% of cases [5]. In suspected cases of IBC, mastitis and cellulitis are usually included in the differential diagnosis. However, in the absence of constituent findings such as fever, elevated white blood cell count, and failure to improve with antibiotics, clinical suspicion leaned toward IBC. According to the literature, advanced tumor stage and relatively young age at the time of discovery can aid in the clinical diagnosis of this condition [4].

CECT findings in cases of IBC tend to feature thickening of the overlying breast skin, lymphadenopathy, osseous metastasis, and the appearance of diffuse tumor infiltration [6]. This case was positive for these findings, as well as extensive bilateral breast and surrounding chest wall involvement seen on CECT. Due to the scarcity of the literature dedicated to CECT findings in IBC, we retrospectively analyzed these CECT findings and compared them to documented magnetic resonance imaging (MRI) findings. CECT findings included diffuse intraparenchymal lesions and thickening of the skin, both of which are often observed in MRI studies of IBC [7]. MRI findings of skin thickening are sensitive for both early- and late-stage IBC [7]. Thus, after correlating positive CECT findings with documented MRI findings, we believe that in the setting of advanced disease, CECT may be of equivalent value in supporting the diagnosis of IBC. This is also supported by the fact that the main identifier for IBC, regardless of image modality, is the inflammatory-appearing changes, although there is no physiologic inflammatory process as stated above [6]. Given that this case suggests that CECT and MRI may have equivalent utility in advanced IBC, due to overlapping findings as compared to the literature, additional clinical research and systematic review may be warranted to confirm this.

While PET-CT is more useful than CECT in determining distant metastasis, it has not proven to be any more efficacious in changing overall prognosis or management [8]. Typical positive findings on PET-CT are hypermetabolic lesions in the breast and overlying dermal hypermetabolic activity [9]. The PET-CT obtained featured diffuse uptake in the regions described on the CECT as metastatic nodules, contributing to the confidence in the diagnosis. Champion et al. showed that PET-CT has a better sensitivity for the detection of locoregional involvement compared to CECT [8]. Although locoregional involvement was visualized on this CECT, this may not be visualized in less-severe cases. Therefore, PET-CT is often recommended in cases where locoregional involvement is not initially visualized on CECT and high clinical suspicion remains.

Mammography has a limited role in the diagnosis of IBC since the majority of cases do not display a discrete mass [2]. Early detection remains an issue, as mammographic screening is suboptimal due to the diffuse nature of the tumor and inflammation obstructing the visualization of structures. New advances in three-dimensional (3D) mammography may prove to be a more sensitive and specific modality in the early diagnosis of breast cancer and improve patient prognosis [10].

On review of pathology, a singular defining histological subtype associated with IBC is not described in the literature. It is linked with a variety of classes including infiltrating ductal carcinoma, lobular carcinoma, small-cell carcinomas, large-cell carcinomas, and medullary carcinomas [11]. The pathological feature of dermal lymphovascular invasion is one of the few hallmarks of IBC. This invasion can result in the obstruction of lymphatic channels, which contributes to the inflamed appearance of the breast [12]. The diffuse lymphatic involvement and subsequent obstruction contribute to the high degree of metastasis in IBC [13]. The literature suggests that extensive lymphatic or dermal lymphatic involvement is reliable in differentiating between IBC and pseudo-IBC, which is important as this changes treatment protocol [14]. According to recent studies, IBC tends to feature estrogen receptor negativity; however, this is not required in the differentiation of a non-IBC invasive cancer [15]. Estrogen receptor negativity often leads to a statistically significant worse prognosis [1]. Human epidermal growth factor receptor-2 (HER2) positivity is higher in cases of IBC compared to non-IBC invasive cancers. Unfortunately, there is currently no positive prognostic correlation for HER2 positivity in IBC [16]. This patient had a triple-positive status, which resulted in an improved prognosis based solely on estrogen receptor positivity.

## Conclusions

Here, we presented a rare case of IBC with the added novelty of an advanced bilateral presentation. This case demonstrated the essential role of imaging in IBC with a newfound utility of CECT in advanced cases. A learning point from this case is that CECT may be a viable alternative to MRI in diagnosing advanced IBC. Additionally, we learned that CECT was equally efficacious in visualizing locoregional involvement in advanced IBC compared to PET-CT. These findings may be applied in settings where more costly modalities such as MRI and PET-CT are not easily accessible.
